# Eckpunkte evidenzbasierter Gesundheitsinformationen

**DOI:** 10.1007/s00103-021-03321-0

**Published:** 2021-04-21

**Authors:** Klaus Koch

**Affiliations:** Ressort Gesundheitsinformation, Institut für Qualität und Wirtschaftlichkeit im Gesundheitswesen (IQWiG), Im Media Park 8, 50670 Köln, Deutschland

**Keywords:** Gesundheitsinformation, Informierte Entscheidung, Empowerment, Gesundheitskompetenz, Qualität, Health information, Informed decision, Empowerment, Health literacy, Quality

## Abstract

Evidenzbasierte Gesundheitsinformationen sollen Menschen durch verlässliche und verständliche Inhalte zu informierten Entscheidungen in Gesundheitsfragen befähigen und dabei ihre persönlichen Ziele und Erwartungen einbeziehen. Um dies zu erreichen, sind bei der Erstellung von Gesundheitsinformationen eine Reihe von Anforderungen zu beachten, die in diesem Beitrag dargestellt werden. Dazu zählen unter anderem eine systematische Recherche, eine begründete Auswahl der geeigneten Evidenz und die Berücksichtigung der aktuellen Evidenz zur Kommunikation von Zahlen, Risikoangaben und Wahrscheinlichkeiten. Bei der Umsetzung müssen zudem die Fragen, Kompetenzen und Nutzungsgewohnheiten der jeweiligen Zielgruppe beachtet werden. Eine Reihe von deutschsprachigen Informationsangeboten zeigt, dass die Umsetzung der Anforderungen auch für die breite Öffentlichkeit praktikabel ist.

## Abstract (en)

Evidence-based health information is intended to empower people to make informed health decisions and include their personal goals and expectations by providing them with neutral and understandable content. To achieve this, a number of requirements must be followed in the production of health information, which are described in this article. These include, among other things, systematic research, a reasoned selection of appropriate evidence, and consideration of current evidence to communicate numbers, risks, and probabilities. Implementation must also take into account the issues, competencies and usage patterns of the groups for which the information is intended. A number of German-language information services demonstrate that implementation of these requirements is feasible for the general public.

## Einleitung

Zur Beantwortung von Gesundheitsfragen nutzen die meisten Menschen mehrere Quellen [1, 2]. Die am häufigsten genannte Informationsquelle ist dabei immer noch die eigene Ärztin bzw. der eigene Arzt. Die Ergebnisse der Studie „Kommunikation und Information im Gesundheitswesen aus Sicht der Bevölkerung – Patientensicherheit und informierte Entscheidung“ (KomPaS) bestätigen aber, dass sämtliche zur Verfügung stehenden privaten und öffentlichen Quellen genutzt werden [1]: Das Spektrum reicht von Gesprächen in der Familie und mit Freunden bis hin zur Nutzung von Medien wie Zeitung, Radio und Fernsehen. Längst ist auch das Internet zu einer gebräuchlichen Quelle für die Suche nach Antworten zu Gesundheitsfragen geworden. Nach KomPaS nutzten 2017 fast 70 % der befragten Erwachsenen das Internet. Im Jahr 2009 waren es noch 36 %.

Die zunehmende Bedeutung des Internets (und der sozialen Medien) hat aktuell auch zu stärkerer Aufmerksamkeit auf die Qualität von Informationen geführt [3, 4]. Allerdings: Man kann bei allen Quellen zum Thema Gesundheit an „gute“ und „schlechte“ Informationen geraten.

In diesem Artikel werden zunächst die wichtigsten Grundsätze bei der Erstellung evidenzbasierter Gesundheitsinformationen dargestellt. Danach wird genauer auf die Anforderungen eingegangen, die bereits in Leitlinien festgehalten wurden. Es werden Beispiele für die Anwendungsbereiche von Gesundheitsinformationen gegeben und es wird darauf hingewiesen, dass auch die Kompetenzen und Nutzungsgewohnheiten der jeweiligen Zielgruppen einbezogen werden müssen. Hinsichtlich der praktischen Umsetzung werden Herausforderungen benannt und wichtige Anbieter von evidenzbasierten Gesundheitsinformationen in Deutschland vorgestellt.

## Was kennzeichnet evidenzbasierte Gesundheitsinformationen?

Das Recht auf umfassende und evidenzbasierte Gesundheitsinformationen, die in verständlicher Weise zur Verfügung stehen, ist im Rahmen des Patientenrechtegesetzes als Anspruch an die (ärztliche) Aufklärung verankert [5]. Evidenzbasierte Gesundheitsinformationen haben das Ziel, Menschen in gesundheitlichen Entscheidungen zu unterstützen. Sie sollen Individuen dazu befähigen, ihren Alltag auch unter gesundheitlichen Aspekten zu gestalten, ihre Erkrankungen gut zu verstehen und die Vor- und Nachteile von Interventionen realistisch einzuschätzen [6]. Da die Präferenzen von Menschen sehr unterschiedlich ausfallen können, stellt dieses Wissen die Voraussetzung für Entscheidungen dar, die im Einklang mit ihren persönlichen Zielen und Erwartungen stehen. Aus Perspektive der Gesundheitsversorgung sind evidenzbasierte Informationen auch deshalb anzustreben, weil sie der Arzt-Patienten-Kommunikation ein solides Fundament geben (Shared Understanding of Medicine), überzogene Erwartungen an die Medizin abbauen und damit auch Unter‑, Über- und Fehlversorgung entgegenwirken könnten [7].

Evidenzbasierte Gesundheitsinformationen basieren auf der Akzeptanz zweier Grundsätze: Wahrung der Selbstbestimmung der informierten Personen und Wissenschaftlichkeit. Beim ersten Grundsatz geht es um die Haltung, Menschen als selbstbestimmte Personen anzuerkennen, also im Sinne des patientenzentrierten Paradigmas auch als Experten für die eigene Gesundheit und Krankheit [8]. Informationsmaterialien sollten sich an den Bedürfnissen von Bürgerinnen und Bürgern bzw. Patientinnen und Patienten orientieren und sie zu selbstbestimmtem Handeln befähigen (Empowerment). Eine evidenzbasierte Gesundheitsinformation will also Menschen nicht in eine bestimmte Richtung drängen. Sie ergänzt die ärztlichen Beratungen und soll beide Seiten zu einer gemeinsamen Entscheidungsfindung befähigen (Shared Decision Making). Das ist in Deutschland für die Informationspflichten von Gesundheitsberufen auch gesetzlich verankert [5]. Diese Haltung schließt ein, die Kompetenzen der Personen zu beachten, für die eine Information gedacht ist. Erhebungen zur Gesundheitskompetenz der Bevölkerung weisen darauf hin, dass große Bevölkerungsgruppen es als schwierig bis unmöglich einschätzen, verlässliche Informationen zu finden, zu verstehen und sie von „schlechten“ Informationen zu unterscheiden [9].

Der zweite Grundsatz bei der Erstellung der Information ist die Haltung, nach wissenschaftlichen Prinzipien vorzugehen. Das betrifft sowohl die Auswahl der Inhalte als auch die Darstellung und Aufbereitung. Die Qualität von Gesundheitsinformationen ist in den letzten Jahrzehnten zu einem eigenen und breiten Forschungsfeld geworden [10, 11]. Aus dieser Forschung leiten sich Eckpunkte für die Erstellung guter Gesundheitsinformationen ab.

Die Erstellung folgt dabei generell den Vorgehensweisen, die in der modernen Medizin überall dabei helfen, Entscheidungen nach dem aktuellen Stand des Wissens zu treffen. Wichtige Grundlage ist die Evidenz – also die sorgfältig recherchierten und bewerteten Ergebnisse aus der (medizinischen) Forschung. Gegenstand aktiver Forschung ist gleichzeitig die Frage, wie man medizinisches Wissen verständlich vermittelt [11]. Auch zu guter Kommunikation gibt es also Evidenz. Evidenzbasierung hat also eine doppelte Bedeutung: Sie bezieht sich zum einen auf die Sachinhalte der Information, zum anderen auch auf die Umsetzung und Kommunikation.

Die Wirkung einer Orientierung an Empowerment und Evidenzbasierung darf nicht unterschätzt werden: Wer sich diesen Zielen verpflichtet, organisiert seinen kompletten Prozess der Erstellung von Gesundheitsinformation entsprechend.

## Anforderungen an evidenzbasierte Gesundheitsinformationen

Einen methodischen Rahmen für die Erstellung von evidenzbasierten Gesundheitsinformationen bieten die Gute Praxis Gesundheitsinformation des Deutschen Netzwerks Evidenzbasierte Medizin e. V. (EbM-Netzwerk; [10]) und die Leitlinie evidenzbasierte Gesundheitsinformation, die in Zusammenarbeit des EbM-Netzwerkes mit der Universität Hamburg entstand [11]. Für die Entwicklung von Entscheidungshilfen hat die International Patient Decision Aid Standards (IPDAS) Collaboration geeignete Empfehlungen erarbeitet [12].

Im Folgenden werden 8 übergreifende Anforderungen an evidenzbasierte Gesundheitsinformationen in Form eines Auszugs aus der Guten Praxis Gesundheitsinformation [10] beschrieben. Die dazu gehörenden Grundsätze sind in Infobox 1 dargestellt. Da das methodische Vorgehen zur Erstellung der Gesundheitsinformation der Fragestellung und den Zielen angemessen sein soll, schreibt die Gute Praxis Gesundheitsinformation Verfassern und Herausgebern nicht vor, welche Methoden und Prozesse verwendet werden müssen. Gefordert wird aber, dass Herausgeber von Gesundheitsinformationen ihr Vorgehen nachvollziehbar darlegen und durch eine transparente Beschreibung der zugrunde liegenden Methoden und Prozesse begründen. Die kann zum Beispiel durch die Veröffentlichung eines frei zugänglichen Methodenpapiers erfolgen, in der die Vorgehensweise beschrieben ist.

Die Anforderungen gemäß [10] sind:

**Identifizierung besonderer Informationsbedürfnisse:** Aus den oben beschriebenen Grundsätzen (Infobox 1) leitet sich die Notwendigkeit ab, sich explizit mit den Bedürfnissen der Nutzerinnen und Nutzer auseinanderzusetzen. Gesundheitsinformationen sollen in Komplexität, Inhalten und Verständlichkeit an die Bedürfnisse und Kompetenzen der Zielgruppe angepasst sein. Zur Erhöhung der Relevanz, Verständlichkeit und Gebrauchstauglichkeit der Gesundheitsinformationen soll die Perspektive von Patientinnen und Patienten bzw. Nutzerinnen und Nutzern in die Entwicklung und Evaluation einer Information einbezogen werden.

**Systematische Recherche: **Grundlage der Erstellung ist eine systematische Recherche der für die Fragestellung angemessenen aktuellen Literatur. Eine systematische Recherche bedeutet nicht zwangsläufig, dass die gesamte medizinische Literatur in mehreren Datenbanken gesucht werden muss. Je nach Fragestellung kann eine Recherche zum Beispiel auf wenige Datenbanken, kurze Zeiträume, wenige Sprachen, bestimmte Studientypen oder Teilfragestellungen eingeschränkt sein. Entscheidend ist, dass der Umfang der Recherche und die Ein- und Ausschlusskriterien den Zielen der Information entsprechen und bei der Darstellung angemessen berücksichtigt werden.

**Auswahl der Evidenz: **Je nach Frage sind die für die Zielgruppe relevanten, vorhandenen wissenschaftlichen Erkenntnisse aus den aktuellen und ausreichend zuverlässigen Studien zu berücksichtigen. Welche Studientypen angemessen sind, hängt von der Fragestellung ab. Unsicherheiten und die Möglichkeit von Verzerrungen (Bias) sind grundsätzlich bei der Erstellung der Gesundheitsinformation zu berücksichtigen. Wenn Unterlagen der „besten“ Evidenzstufe fehlen, schließt das die Erstellung von evidenzbasierten Gesundheitsinformationen nicht aus. Dann kommt es aber darauf an, die Unsicherheiten angemessen wiederzugeben.

**Auswahl der dargestellten Ergebnisse (Krankheitsfolgen): **Informationen über Krankheiten und Behandlungsergebnisse sollen sich auf Aspekte der Gesundheit beziehen, die für Patientinnen und Patienten relevant sind, also insbesondere auf die Sterblichkeit (Mortalität), die Beschwerden und Komplikationen (Morbidität), die gesundheitsbezogene Lebensqualität und die Begleitumstände der Behandlung (z. B. Zeitaufwand, körperliche, seelische, soziale und auch finanzielle Belastungen). In Studien werden diese Aspekte „Endpunkte“ genannt, weil sie das Ziel der Untersuchung sind. Liegen zu relevanten Endpunkten keine ausreichend verlässlichen Ergebnisse vor, ist dieser Umstand anzusprechen. Wenn nur Ersatzendpunkte (Surrogatparameter, Biomarker) wie etwa Blutwerte zum Beispiel zur Beschreibung von Effekten einer Therapie herangezogen werden, ist auf die sich daraus ergebenden Unsicherheiten hinzuweisen.

**Wahl und Darstellung von Vergleichen: **Für die individuelle Abwägung von Nutzen und Schaden einer medizinischen Maßnahme (Intervention) sind Informationen über die patientenrelevanten Ergebnisse im Vergleich zu einer Scheinintervention (zum Beispiel Placebo) oder zu einer anderen Behandlungsoption erforderlich. Eine Information sollte auch beinhalten, welche Ergebnisse zu erwarten sind, wenn auf eine Behandlung verzichtet wird.

**Umgang mit Zahlen und Risikoangaben: **Auch für Behandlungen mit einem nachgewiesenen Nutzen gilt, dass nicht bei allen Patientinnen oder Patienten positive Effekte eintreten, manche erleiden sogar einen Schaden. Deshalb ist eine ausgewogene Darstellung des möglichen Nutzens und Schadens einer Intervention erforderlich.

**Berücksichtigung von Alters- und Geschlechterunterschieden**: Der natürliche Krankheitsverlauf, die Risiken, Symptome, Morbidität, Mortalität, Wirkungen und unerwünschte Wirkungen von Interventionen, die gesundheitsbezogene Lebensqualität und die Begleitumstände einer Behandlung können je nach Alter oder Geschlecht variieren. Sofern es bedeutsame Unterschiede gibt, sollen diese bei der Erstellung einer Information berücksichtigt werden.

**Transparenz über Verfasser und Herausgeber:** Angaben über Verfasser und Herausgeber und ihre Finanzierung sollen transparent sein. Pflichtangaben über den Herausgeber einer Information sind durch rechtliche Vorgaben geregelt, die jeder Herausgeber einhalten muss. Um Transparenz zu schaffen, sind aber weitergehende Angaben nötig (wie etwa Angaben über die Finanzierung eines Angebots, die Qualifikation der Verfasser, mögliche Interessenkonflikte etc.).

## Wann werden evidenzbasierte Gesundheitsinformationen benötigt?

Menschen benötigen evidenzbasierte Gesundheitsinformationen vor allem, wenn sie vor präferenzsensitiven Entscheidungen stehen – also solchen, die von Person zu Person unterschiedlich ausfallen können [13]. Unterschiedliche Präferenzen können dabei viele verschiedene Gründe haben. Dazu gehören unterschiedliche Ziele, Prioritäten, Erwartungen und Kontexte. Die Handlungsalternativen können unterschiedliche Vor- und Nachteile haben. Beispiele für präferenzsensitive Entscheidungen werden in Büchter und Albrecht (2019) benannt [13]^1^:

Individuelle Behandlung:

- Die Entscheidung für eine konservative Behandlung oder Rekonstruktion bei einem isolierten Riss des vorderen Kreuzbands
- Die Entscheidung zwischen aktiver Überwachung, Strahlentherapie und Operation bei der Behandlung des Niedrig-Risiko-Prostatakrebs
- Die Entscheidung für oder gegen eine vaginale Geburt nach Kaiserschnitt
- Die medikamentöse Behandlung von Risikofaktoren wie einer erniedrigten Knochendichte, ungünstigen Cholesterinwerten oder Bluthochdruck

Angebote zur Prävention:

- Die (Nicht‑)Teilnahme an Früherkennungsuntersuchungen
- Die Entscheidung für oder gegen einen Organspendeausweis
- Die Entscheidung für oder gegen Lebensstiländerungen zur gesundheitlichen Prävention

Daneben können Gesundheitsinformationen auch andere, nicht minder wichtige, Ziele verfolgen. Dazu gehören zum Beispiel:

- Die Vermittlung von allgemeinem Wissen zu einer Erkrankung, zum Beispiel Beschwerden, Verlauf, Behandlung und Vorbeugung.
- Die Klärung praktischer Versorgungsfragen (Wie beantrage ich eine kardiovaskuläre Rehabilitation?)
- Die Vermittlung von Bewältigungsstrategien im Leben mit einer chronischen Erkrankung (Wie gehen andere Menschen mit Schuppenflechte im Beruf mit ihrer Erkrankung um?)
- Die Unterstützung von Angehörigen (Wie gehe ich auf jemanden zu, der ein Alkoholproblem hat?)

Dabei ist es immer sinnvoll, Informationsangebote so zu gestalten oder einzubetten, dass sie Betroffenen auch emotionale Unterstützung bieten können.

### Herausforderungen bei der praktischen Umsetzung

Bei der Umsetzung der oben beschriebenen Anforderungen ist es wichtig, die unterschiedlichen Kompetenzen und Nutzungsgewohnheiten der Menschen zu beachten. Eine wissenschaftliche Herangehensweise an die Erstellung von Gesundheitsinformationen birgt das Risiko, andere Qualitätsdimensionen, wie zum Beispiel Gestaltung oder Umfang einer Information, zu unterschätzen. Das liegt auch daran, dass die Forschung zur Qualität von Gesundheitsinformationen sich bislang auf Dimensionen, wie zum Beispiel die Vermittlung von Risiken und Unsicherheiten, fokussiert hat.

Die oftmals idealtypisch dargestellten Anforderungen an evidenzbasierte Gesundheitsinformationen erfordern in der Praxis deshalb oft Abwägungen, bei denen auch Aspekte eines angemessenen (bzw. unterschiedlichen Gruppen von Leserinnen und Lesern zumutbaren) Umfangs, der Gestaltung und der Barrierefreiheit zu berücksichtigen sind.

Da kein Informationsangebot gleichzeitig für alle Bevölkerungsgruppen optimiert sein kann, ist zudem eine gewisse Aufgabenteilung nötig: Ideal wäre es, wenn sich zum Beispiel in Deutschland die Anbieter von evidenzbasierten Informationen verständigen würden, welche sich ergänzenden Schwerpunkte und Spezialisierungen sinnvoll sind. Diese Verständigung könnte zum Beispiel unter dem Dach eines nationalen Gesundheitsportals stattfinden (siehe Infobox 2).

In der Umsetzung erfordern evidenzbasierte Gesundheitsinformationen deshalb eine Verbindung aus wissenschaftlichen, redaktionellen und gestalterischen Kompetenzen. Wichtig ist zudem, schon bei der ersten Erstellung einer Information den Bedarf für kommende Aktualisierungen einzuplanen. Herausgeber, die die Anforderungen an evidenzbasierte Informationen ernsthaft umsetzen wollen, müssen deshalb in der Regel zusätzliche Kompetenzen und Ressourcen einplanen.

Bei der Umsetzung können durchaus Konflikte entstehen. Erfahrungen zeigen, dass auch in Institutionen und Behörden nicht von vornherein ein Verständnis für die Qualität evidenzbasierter Gesundheitsinformationen vorausgesetzt werden kann. Evidenzbasierte Informationen kollidieren zudem immer wieder mit Marketinginteressen oder schließen sich (im Falle von Produktwerbung) meist gegenseitig aus. Das führt dazu, dass evidenzbasierte Informationen in der Regel eine gemeinnützige Finanzierung benötigen.

Ferner stellt auch eine gute Umsetzung der Kriterien nicht automatisch sicher, dass sich die erstellten Informationen im Wettbewerb mit anderen (und schlechteren oder gezielt irreführenden) Angeboten durchsetzen. Daher sollten Herausgeber schon bei der Planung evidenzbasierter Informationen Implementierungs- oder Disseminationsstrategien dafür entwickeln, wie ihre Informationen gefunden und breit genutzt werden können.

### Bereits existierende Angebote

Bislang existiert in Deutschland kein allgemein akzeptiertes Instrument zur Qualitätsbewertung von Gesundheitsinformationen in den verschiedenen Formaten. In Infobox 2 sind ausgewählte Angebote von Herausgebern genannt, die Methodenpapiere veröffentlicht haben, in denen beschrieben wird, nach welchen Prozessen und Methoden die Herausgeber ihre Gesundheitsinformationen erstellen. Die Beispiele zeigen, dass die Umsetzung der Anforderungen auch für die breite Öffentlichkeit praktikabel ist.

## Fazit

Das Recht auf umfassende und evidenzbasierte Gesundheitsinformation, die in verständlicher Weise zur Verfügung stehen, ist im Rahmen des Patientenrechtegesetzes als Anspruch an die (ärztliche) Aufklärung verankert. Insbesondere für öffentlich finanzierte Informationsangebote ergibt sich daraus ganz unmittelbar ein ethischer Anspruch an die Erstellung von Gesundheitsinformationen und ganz allgemein an die Kommunikation zu Gesundheitsfragen. Dieser Beitrag skizziert, dass für die Umsetzung dieses Anspruchs im deutschsprachigen Raum bereits Hilfestellungen wie die „Gute Praxis Gesundheitsinformation“ und die „Leitlinie evidenzbasierte Gesundheitsinformation“ existieren, an denen sich Herausgeber orientieren können. Eine Reihe von deutschsprachigen Informationsangeboten zeigt, dass dieser Anspruch grundsätzlich auch für die breite Öffentlichkeit umsetzbar ist.

Gleichzeitig besteht aber Bedarf, die bestehenden Angebote weiterzuentwickeln. Nötig ist zum Beispiel, die Breite und Tiefe der Angebote sowie die genutzten Kommunikationskanäle in Deutschland zu erweitern, auch für Gruppen mit unterschiedlichen Kompetenzen und Gesundheitsfragen. Das erfordert eine institutionsübergreifende Kooperation und Koordination. Diese Koordination könnte unter dem Dach eines nationalen Gesundheitsportals stattfinden. Welchen Platz das Portal im deutschen Informationsangebot haben und wie es ausgestaltet werden kann, hängt von der derzeit stattfindenden Klärung grundsätzlicher Fragen ab.

### Infobox 1 Grundsätze evidenzbasierter Gesundheitsinformationen [10]

- Systematische Recherche entsprechend der für die Zielgruppe relevanten Fragestellungen
- Begründete Auswahl der für die Fragestellung geeigneten Evidenz
- Unverzerrte Darstellung der für Patientinnen und Patienten relevanten Forschungsergebnisse, zum Beispiel zu Sterblichkeit (Mortalität), Beschwerden/Komplikationen (Morbidität) und gesundheitsbezogener Lebensqualität
- Angemessene inhaltliche und sprachliche Darstellung von Unsicherheiten
- Entweder Verzicht auf direktive Empfehlungen oder klare Trennung zwischen der Darstellung von Ergebnissen und der Ableitung von Empfehlungen
- Berücksichtigung der aktuellen Evidenz zur Kommunikation von Zahlen, Risikoangaben und Wahrscheinlichkeiten
- Transparente Darstellung der Angaben über Verfasser und Herausgeber der Gesundheitsinformation und deren Finanzierung

### Infobox 2 Anbieter von evidenzbasierten Gesundheitsinformationen

Das gemeinnützige *Institut für Qualität und Wirtschaftlichkeit im Gesundheitswesen (IQWiG)* hat den gesetzlichen Auftrag, alle Bürgerinnen und Bürger allgemeinverständlich und evidenzbasiert über einen breiten Katalog medizinischer Themen zu informieren: www.gesundheitsinformation.de.

Das *Ärztliche Zentrum für Qualität in der Medizin*, eine gemeinsame Einrichtung von Bundesärztekammer und Kassenärztlicher Bundesvereinigung, veröffentlicht unter anderem Kurzinformationen und Patientenleitlinien: www.patienten-information.de.

Der *Krebsinformationsdienst des Deutschen Krebsforschungszentrums (DKFZ)* ist Ansprechpartner für alle Fragen zum Thema Krebs: www.krebsinformationsdienst.de. Er bietet auch telefonische Beratung.

Der *medizinische Dienst des Spitzenverbands der Krankenkassen* gibt eine Bewertung individueller Gesundheitsleistungen heraus: www.igel-monitor.de.

Die *gemeinnützige Stiftung Gesundheitswissen* veröffentlicht evidenzbasierte Gesundheitsinformation zu einer wachsenden Zahl von Themen: www.stiftung-gesundheitswissen.de.

Das *Bundesgesundheitsministerium* baut seit September 2020 unter der Adresse gesund.bund.de ein „nationales Gesundheitsportal“ auf, das mit den oben genannten Angeboten des IQWiG und DKFZ kooperiert.

## Footnotes

1 Wörtlich zitiert nach [13].
